# Appearance of aldehydes in the surface layer of lake waters

**DOI:** 10.1007/s10661-014-3720-y

**Published:** 2014-03-28

**Authors:** Agata Dąbrowska, Jacek Nawrocki, Elżbieta Szeląg-Wasielewska

**Affiliations:** 1 Department of Water Treatment Technology, Faculty of Chemistry, A. Mickiewicz University, ul. Umultowska 89B, 61-614 Poznań, Poland; 2 Department of Water Protection, Faculty of Biology, A. Mickiewicz University, ul. Umultowska 89, 61-614 Poznań, Poland

**Keywords:** Aldehydes, Total organic carbon, Surface water, Lakes, Precipitation

## Abstract

The paper presents results concerning the changes in the content of aldehydes in samples of lake water collected near the lake surface. The study of lake waters was undertaken to explain which physicochemical parameters of the environment have the greatest influence on the level of aldehydes, which of the aldehydes are most often met in surface water and in what concentrations. We observed that formaldehyde, acetaldehyde, propanal, glyoxal, methylglyoxal and acetone were commonly present in surface water samples, while semi-volatile and poorly soluble aldehydes such as nonanal and decanal were observed seasonally. The contents of total aldehydes varied in a wide range, from 55 to 670 μg/l, and the concentration of total organic carbon varied significantly from 3 to 18 mg /l, but there was no evident correlation between them in all of samples. The total content of aldehydes did not depend on the meteorological parameters such as air temperature, UV radiation and ozone concentration; however, it was noted that the level of carbonyl concentration is related to the period of intense precipitation: in the period of very low precipitations, the highest contents of total aldehydes were determined in all of the water samples, and in the periods of intense precipitations, the content of total aldehydes was drastically smaller.

## Introduction

Aldehydes are commonly found in the environment. Their presence can be controversial as they belong to the group of compounds potentially charged with considerable toxicity and carcinogenic and mutagenic properties (Richardson et al. 2007; Hebert et al. 2010; Rice et al. 2012). Aldehydes can cause an undesirable odour in natural surface waters (Nijssen et al. 1996; Bao et al. 1997). They can originate from a number of sources related to the oxidation processes, photochemical transformations, life processes of animals and plants and decomposition of organic matter. A significant part of aldehydes are formed in the atmosphere and their main precursors are organic compounds of natural or anthropogenic origin. The reaction of the commonly present oxidants (such as ozone, hydrogen peroxide or hydroxyl radicals) with the hydrocarbons has an important influence on the formation of carbonyl compounds in the troposphere (Warneck 2005; Possanzini et al. 2007; Myriokefalitakis et al. 2008; Obermeyer et al. 2009).

Another natural source of aldehydes is their emission from the plants, related to the vegetation processes (Wildt et al. 2003; Geron et al. 2006; Hu et al. 2008, 2011). As suggested by Hu et al. (2008, 2011), aldehydes can be released from plants in response to the environmental stress imposed by pests eating plant leaves, diseases caused by fungi parasites or to repel the competitors. Hu and co-workers have identified more than ten aldehydes emitted by plants, including the saturated aldehydes such as acetaldehyde and C4–C13, the unsaturated ones—hexanal and nonenal, benzaldehyde and furfural. The aldehydes accumulating in the atmosphere are periodically removed from it by dry deposition with falling dust or by wet deposition—with precipitations (rain, snow, hail or fog) (Kawamura et al. 2000, 2001; Matsunaga and Kawamura 2000; Matsunaga et al. 2007). The precipitations have been checked for the presence of organic and inorganic pollutants. Formaldehyde, acetaldehyde, glyoxal, methylglyoxal and acetone are the carbonyls most often determined in rain and described in literature (Matsunaga and Kawamura 2000; Kawamura et al. 2001; Matsumoto et al. 2005; Matsunaga et al. 2007; Li et al. 2008; Basheer et al. 2010). These aldehydes can also be present in surface waters. They can be formed in the processes of oxidation, photochemical transformations, living processes of microorganisms and water vegetation, so in similar processes as in the atmosphere. Jalliffier-Merlon et al. (1991) pointed to the participation of phytoplankton in the production of aldehydes C_6_ to C_13_ at the mouth of the Rhône River. They noted a strong correlation between the phytoplankton biomass expressed as the content of *a* chlorophyll and the total concentration of aldehydes. The same authors reported a decrease in the aldehyde concentration with increasing water temperature, which they explained to be caused by the high volatility of aldehydes and an increase in intensity of biodegradation processes in summer. They suggested the use of aldehydes as biomarkers of biological life in surface waters, e.g. an increased concentration of nonanal can indicate the presence of hardly detectable blue algae (*Cyanophyceae*). Hammes et al. (2007) observed an effect of the phytoplankton on a significant increase in the content of aldehydes, ketones and carboxylic acids in the water collected from Lake Zurich and treated by ozone.

The presence of aldehydes in surface waters has been rather poorly recognised in literature. It has not been established whether the content of aldehydes in surface waters is related only to the conditions of biological life and transformation of organic matter in the water or it also depends on the external factors and deposition of carbonyl compounds from the atmosphere. The systematic study of the near-surface layer of lake water was undertaken to explain if the content of aldehydes depends on the atmospheric conditions, which of the aldehydes are most often met in surface water and in what concentrations and which physicochemical parameters of the environment have the greatest influence on the level of aldehydes.

This paper presents results of the aldehyde determination in water samples collected from the surface layer of a few lakes and their relation with the amount of precipitations, season of the year, concentration of ozone, intensity of UV radiation and content of total organic carbon and particulate matter suspended in the waters (defined as seston). The analysis of aldehydes was performed by the method proposed by the US Environmental Protection Agency (Method 556.1, Revision 1.0, September 1999) with a preliminary derivatisation process and separation of the created oxymes by gas chromatography in the configuration with electron capture detector.

## Materials and methods

### Collection of natural water samples

Water samples were collected from six lakes situated in Poland (near Poznań City). The lakes are characterised in Table 1. The samples were collected at sampling stations, several times during the year (2010–2012); relative to the year seasons, the stations were situated in the profundal zone, i.e. the deepest site in the lake. The 500-ml water samples were collected from the near-surface layer, at depths no greater than 0.5 m.

**Table 1:** Lake description

Name of Lake	Lake location—link to the corresponding maps	Area [km^2^]	Maximum depth [m]
Góreckie	Strictly protected area of the Wielkopolski National Park; http://maps.google.pl/maps?hl=pl&ie=UTF8&ll=52.257546,16.790886&spn=0.080909,0.155697&t=m&z=13&vpsrc=6	1.00	17
Strzeszyńskie	http://maps.google.pl/maps?hl=pl&ie=UTF8&ll=52.462207,16.826077&spn=0.020134,0.038924&t=m&z=15&vpsrc=6	0.32	18
Sławskie	http://maps.google.pl/maps?hl=pl&ie=UTF8&ll=51.867376,16.045532&spn=0.163237,0.311394&t=m&z=12&vpsrc=6	8.00	12
Uzarzewskie	http://maps.google.pl/maps?hl=pl&ie=UTF8&t=m&vpsrc=6&ll=52.448373,17.133694&spn=0.04028,0.077848&z=14	0.11	7
Swarzędzkie	http://maps.google.pl/maps?hl=pl&ie=UTF8&ll=52.415247,17.067261&spn=0.04031,0.077848&t=m&z=14&vpsrc=6	0.79	7
Maltańskie	Artificial lake; http://maps.google.pl/maps?hl=pl&ie=UTF8&ll=52.403047,16.97319&spn=0.040321,0.077848&t=m&z=14&vpsrc=6	0.64	3

Ozone concentration, UV_B_ radiation, temperature and level of precipitation were measured systematically in an automatic system by the Provincial Inspectorate of Environmental Protection in Poznań (consistently with Council Directive 93/62/EC on ambient air quality assessment and management).

Since aldehydes are easily biodegradable, the aqueous samples should be analysed as soon as possible. Thus, the samples were typically derivatized on the day of their collection. If it was impossible, the samples were protected from biodegradation by addition of 25 mg of copper sulphate per 50 ml of water and stored at a temperature of 4 °C. If necessary, the samples were filtered through glass fibre filter GF/F (Whatman, Schleicher&Schuell, UK) to remove suspended matter. Formaldehyde and acetaldehyde are typically present in the laboratory air; thus, care was taken to minimize the exposure of reagents and the sample water to the air. Glassware was scrupulously cleaned by detergent, washed with hot water, rinsed with free organic water and finally dried. After cleaning, the glassware was stored in a clean environment to prevent any accumulation of dust or other contaminants.

### Chemicals

The compounds *O*-(2,3,4,5,6-pentafluorobenzyl)hydroxylamine (PFBOA) and analytical standards (aldehydes and ketones) were purchased from Aldrich-Chemie (Steinheim, Germany) and BDH (Pool, UK; AnalaR and GPR grades). PFBOA was prepared gravimetrically as an aqueous solution (2 mg/ml) in organic free water. Aldehyde and ketone solutions were prepared gravimetrically in methanol (J. T. Baker, Germany) from pure compounds. GC-grade *n*-hexane (J. T. Baker, Germany) was used as a solvent for extraction. Sulphuric acid and copper sulphate (Promochem, Poland) were of analytical grade.

### Aldehyde analysis

The high polarity and reactivity of carbonyl compounds in water matrices imposes the need for their derivatization as the derivatives are less polar, more volatile and can be detected using selective detectors. PFBOA is recommended by the US Environmental Protection Agency as a derivatizing agent. The technique uses direct aqueous derivatization with PFBOA reagent, which reacts with the aldehydes to form the corresponding oximes. With most of the aldehydes, two geometric isomers are formed: E- and Z-PFBO, except for symmetrical carbonyls such as formaldehyde or acetone. The oximes were extracted by shaking the solution with 1 ml of hexane and analysed by gas chromatography using GC 8000 series (Fisons Instruments) equipped with ^63^Ni electron capture detector. The Rtx-5MS (Restek) fused silica capillary column (30 m × 0.25 mm i.d. × 0.25 μm film) was applied for separation.

A list of the studied carbonyl compounds and their detection limits is presented in Table 2. The details of aldehyde analysis were described by us earlier (Dąbrowska et al. 2003a, 2005; Jeleń et al. 2004).

**Table 2:** List of studied compounds, linearity of calibration curve and limit of detection

Compound	Linear formula	Molecular weight	Calibration curve	Relative standard deviation	
		[g/mol]	Measurement range [μg/l]	Linearity, *R* ^2^	[%]
Methanal formaldehyde	HCHO	30.03	4–150	0.991	9.8
Ethanal acetaldehyde	CH_3_CHO	44.05	4–150	0.995	9.1
Propanal propionalaldehyde	CH_3_CH_2_CHO	58.08	2–100	0.998	8.2
Butanal butyraldehyde	CH_3_(CH_2_)_2_CHO	72.11	2–100	0.997	8.5
Pentanal valeraldehyde	CH_3_(CH_2_)_3_CHO	86.13	2–100	0.997	6.2
Hexanal caproaldehyde	CH_3_(CH_2_)_4_CHO	100.16	2–100	0.998	6.4
Heptanal enanthaldehyde	CH_3_(CH_2_)_5_CHO	114.19	2–100	0.998	6.8
Octanal caprylic aldehyde	CH_3_(CH_2_)_6_CHO	128.21	2–100	0.997	7.9
Nonanal pelargonaldehyde	CH_3_(CH_2_)_7_CHO	142.24	2–100	0.999	8.7
Decanal caprinalaldehyde	CH_3_(CH_2_)_7_CHO	156.20	2–100	0.996	8.5
Benzaldehyde benzoic aldehyde	CH_3_(CH_2_)_8_CHO	106.12	2–100	0.996	7.5
Glyoxal oxalaldehyde	H(C = O)CHO	58.04	0.1–100	0.998	6.8
Methylglyoxal pyruvaldehyde	CH_3_(C = O)CHO	72.06	0.1–100	0.997	6.9
Propanone acetone	CH_3_COCH_3_	58.08	1–100	0.991	8.6

### TOC, seston and chlorophyll *a* measurements

Total organic carbon (TOC) in selected aqueous samples was measured by means of AURORA Model 1030 (I.O. Analytical) using the persulphate/100 °C wet oxidation method. The amount of carbon dioxide was measured with IR detector. The method detection limit was 0.01 mg C/l; relative standard deviation (RSD) of the method was 3 %.

Suspended solids were weighted after filtration through Whatman GF/F glass fiber filters and desiccation at 105 °C. The amount of seston was expressed as dry weight. Chlorophyll *a* concentrations were determined by the spectrophotometric method based on 0.5–1-l samples filtered through Whatman GF/F glass fiber filters. Pigments were extracted with 90 % acetone during 24 h in the dark at 4 °C, and the calculations were carried out using Lorenzen’s formula.

## Results and discussions

The samples were collected near the surface layer of lake water and at different depths along the profile at the profundal zone. This paper presents the first part of our results concerning the qualitative and quantitative changes in the content of aldehydes in samples of surface waters. Table 3 shows the concentration of aldehydes and total organic carbon measured in water samples. Aldehydes were found in all of the lakes studied, irrespective of their area or maximum depth. The total concentrations of aldehydes determined in the samples varied from 55 to 674 μg/l, while the mean values (*n* = 64) varied from 167 to 324 μg/l. The considerable changes in aldehyde concentrations can suggest the influence of different environmental factors on the formation of carbonyl compounds. A very important source of aldehyde origin can be organic matter and its transformation taking place in natural waters. In the samples collected from the lake surface, the concentration of total organic carbon varied from 3 to 18 mg/l, while the mean values varied from 6 to 12 mg/l. The highest mean concentration of total aldehydes in the near-surface layer was measured in Lake Góreckie and amounted to over 320 μg/l. The content of total organic carbon in this lake was also higher than in the other lakes (mean value 12 mg/l). The water from Lake Góreckie is characterised by high biological productivity in spite of the fact that some point sources of pollution have been eliminated and the area surrounding this lake has been taken under strict protection. In the water of this lake, a high correlation (*r* = 0.88) was found between the content of total aldehydes and the content of total organic carbon. A high correlation coefficient (that is *r* = 0.71) between these two parameters was established also in the water of Lake Sławskie of the largest area from among the lakes studied. In this lake, the mean content of total aldehydes was 167 μg/l , while the mean content of total organic carbon was 7 mg/l. In the water from the other lakes studied, no such correlation was found. The amount of carbonyl compounds produced as a result of different physicochemical processes taking place in water is not directly related to the amount of natural organic matter but to its quality (Dąbrowska et al. 2003b). The mean percentage contribution of the aldehydes monitored in the content of total organic carbon was close to 2 % and varied from 1.32 to 2.88 %. The lowest contribution of aldehydes in TOC was noted in the water of the artificial Lake Maltańskie.

**Table 3:** Concentration of aldehydes and total organic carbon in surface water samples

Name of lake	Góreckie (*n* = 12)	Sławskie (*n* = 10)	Strzeszyńskie (*n* = 10)	Swarzędzkie (*n* = 10)	Uzarzewskie (*n* = 14)	Maltańskie (*n* = 8)
Range of total aldehyde concentration [μg/l]	82–642	69–409	55–435	71–538	80–674	93–497
Average total aldehyde concentration [μg/l]	324	167	180	286	265	186
Range of total organic carbon [mg/l]	8.17–18.26	3.10–10.36	3.02–9.00	4.11–10.40	5.84–13.10	4.95–12.02
Average total organic carbon [mg/l]	11.63	5.79	5.67	6.94	8.40	8.26

The aliphatic aldehydes from C1 to C10 as well as benzaldehyde, glyoxal and methylglyoxal were determined in all collected samples. Based on the analysis of their concentrations and the frequency of their occurrence (see the data in Table 4), the contributions of formaldehyde, acetaldehyde and propanal were the highest. These three aldehydes were identified in all samples, on average at a level of several tens of micrograms per litre, and their total content made almost 50 % of the content of all aldehydes identified in the samples. The presence of formaldehyde, acetaldehyde and propanal in all collected samples means that these low carbon aliphatic aldehydes commonly occur in the surface layer of lake water, irrespective of the year season and environmental conditions. It is important to stress that along with the three aldehydes cited, acetone was also found in all samples, on average at a concentration of 23 μg/l. Acetone is common in the atmosphere and in many plant tissues (Copeland et al. 2012). This ketone is efficiently produced by the bacteria *Clostridium acetobutylicum*. The mean concentration of formaldehyde and acetaldehyde monitored in the surface layer of Lake Góreckie was much higher than in the other lakes. Lake Góreckie is localised within the strictly protected area of the Wielkopolski National Park, so it can be reasonably assumed that the aldehydes identified in its water are mostly of natural origin. Some aldehydes such as hexanal, heptanal, nonanal and decanal were detected periodically. The seasonal presence of aldehydes is explained by some authors as caused by the vegetation processes taking place in plants as well the seasonal appearance of phytoplankton (Yokouchi et al. 1990; Jalliffier-Merlon et al. 1991; Bowman et al. 2003; Hammes et al. 2007). According to the suggestion of Jalliffier-Merlon et al. (1991), an increase in nonanal concentration indicates the growth of *Cyanophyceae* (blue algae) in surface waters.

**Table 4:** Aldehyde and acetone concentrations and their frequency of occurrence in monitored lake waters

Compounds	Name of lake																	
	Góreckie (*n* = 12)			Sławskie (*n* = 10)			Strzeszyńskie (*n* = 10)			Swarzędzkie (*n* = 10)			Uzarzewskie (*n* = 14)			Maltańskie (*n* = 8)		
	Concentration [μg/l]		Frequency of occurrence [%]	Concentration [μg/l]		Frequency of occurrence [%]	Concentration [μg/l]		Frequency of occurrence [%]	Concentration [μg/l]		Frequency of occurrence [%]	Concentration [μg/l]		Frequency of occurrence [%]	Concentration [μg/l]		Frequency of occurrence [%]
	Range	Average		Range	Average		Range	Average		Range	Average		Range	Average		Range	Average	
Formaldehyde	8.89–122	72.6	100	6.14–80.0	36.6	100	5.78–85.0	43.7	100	15.1–155	58.1	100	4.44–157	62.8	100	14.7–91.1	40	100
Acetaldehyde	14.7–112	53.8	100	6.36–81.2	27	100	6.16–93.8	24	100	5.76–88.3	40.2	100	7.52–72.3	32.5	100	8.72–77.0	30.3	100
Acetone	3.85–57.9	21.4	100	3.85–61.5	23.4	100	5.38–36.9	19	100	3.92–96.2	34	100	7.15–63.9	20.6	100	3.38–58.8	18.4	100
Propanal	7.25–80.6	32	100	7.44–89.3	26.6	100	7.50–128	34.7	100	6.04–90.8	41.5	100	18.3–211	51.6	100	8.46–102	36.2	100
Butanal	3.20–24.4	8.14	92	1.76–7.60	4.56	90	2.00– 19.2	6.86	100	2.08–11.2	7.12	90	3.37–26.9	10	93	2.93–25.9	7.69	88
Pentanal	6.06–29.7	13.5	92	0.10–6.2	1.6	60	4.03–13.3	7.7	60	3.19– 17.8	9.54	70	3.94–40.0	12.3	93	5.28–30.3	10.4	75
Hexanal	4.49–51.5	17	100	2.34–9.02	4.95	100	2.88–74.9	17	80	4.83–52.2	22	90	4.20–87.0	22.6	86	9.02–28.5	15.5	75
Heptanal	6.17–113	33	92	4.94–18.9	12	30	7.00–30.8	14.6	50	6.11–96.7	43.9	60	5.17–17.8	11.94	43	nd		
Octanal	3.21–33.0	13	83	2.50–10.4	6.43	20	6.07–13.2	6.43	20	8.57–8.75	8.66	20	4.72–4.72	4.72	7	nd		
Benzaldehyde	2.18–18.3	7.39	67	10.8–10.8	10.8	10	2.21–2.21	2.21	10	16.4–16.6	16.5	30	6.42–16.7	11.5	14	nd		
Nonanal	1.25–72.4	27.4	83	2.50–73.3	14.4	100	4.00–15.8	9.6	50	1.67–30.0	11.1	80	1.67–50.1	16.9	100	2.84–82.9	18.9	75
Decanal	1.93–49.1	20.8	83	3.71–58.9	15.6	70	3.76–73.9	23.1	40	8.36–19.4	11.9	40	3.21–41.9	13.5	50	2.64–13.9	7.3	75
Glyoxal	0.64–33	11	100	0.10–19	6	90	1.24–10.9	4.26	80	0.55–58	16.6	90	1.74–47.6	19.7	86	0.26–43	10.8	88
Methylglyoxal	0.11–20	7.2	100	3.64–22.7	9.35	60	0.95–18.4	7.27	89	0.93–91	21	80	0.72–40	15.1	64	1.02–19.7	6.17	100

**Fig. 1:**
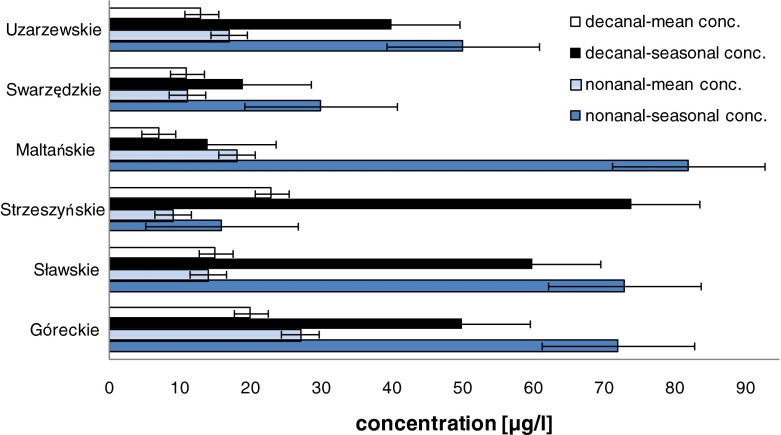
Comparison of mean and seasonally high concentrations of nonanal and decanal identified in lake waters

**Fig. 2:**
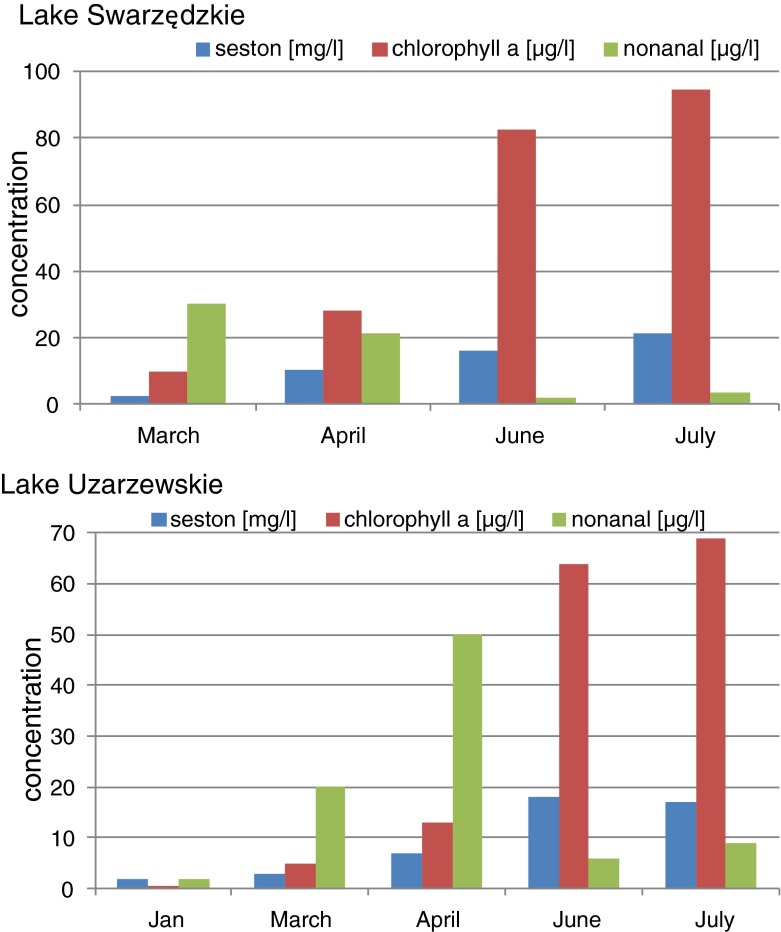
Seasonal changes in the contents of seston, chlorophyll *a* and nonanal in the water samples taken from Lake Swarzędzkie and Lake Uzarzewskie

We observed the irregular appearance of nonanal or decanal in unexpectedly high concentration mostly in spring or in summer, hence in the period of intense vegetation processes. In the samples collected from the surface layer of Lake Góreckie in the spring and summer months (March–July), nonanal was found in concentrations of above 70 μg/l and decanal in concentrations above 50 μg/l. In the same period, a high level of decanal was identified in the near-surface layer of Lake Sławskie and Lake Strzeszyńskie and amounted to above 60 and 70 μg/l, respectively. The high concentration of nonanal was identified also in Lake Maltańskie and Lake Uzarzewskie at 80 and 50 μg/l, respectively. The seasonal elevated concentrations of nonanal and decanal determined in the lake waters are presented in Fig. 1. There is a significant difference between the highest concentration of nonanal and decanal identified in spring with reference to their mean year-long values. Similarly, the concentrations of seston and chlorophyll *a* in the year changed significantly, and they were the highest in the summer. Figure 2 illustrates seasonal changes in the contents of seston, chlorophyll *a* and nonanal in the water samples taken from Lake Swarzędzkie and Lake Uzarzewskie. A maximum concentration of nonanal has been noted in March and April; the highest value of suspended particulate matter as well as chlorophyll has been observed in June and July.

**Fig. 3:**
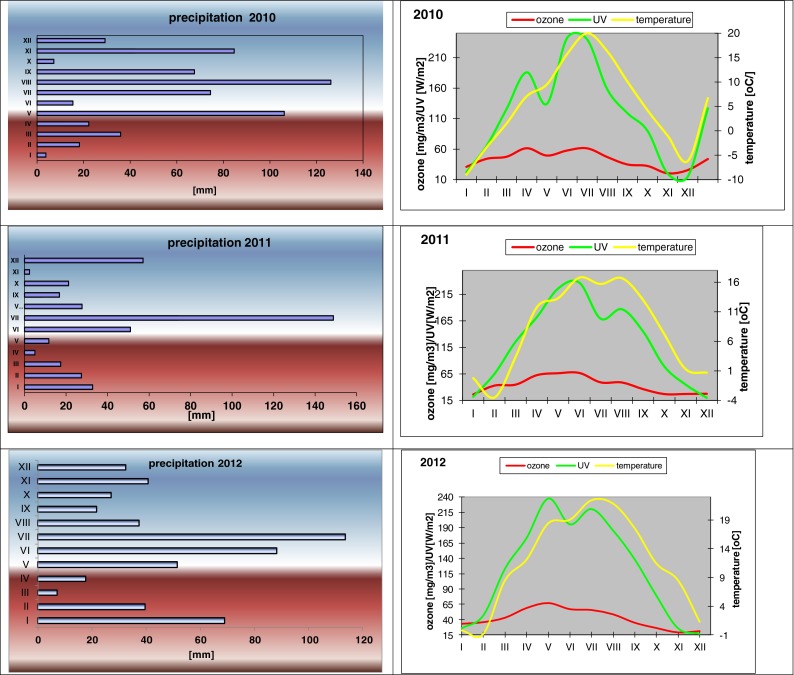
Atmospheric conditions monitored in years 2010–2012

**Fig. 4:**
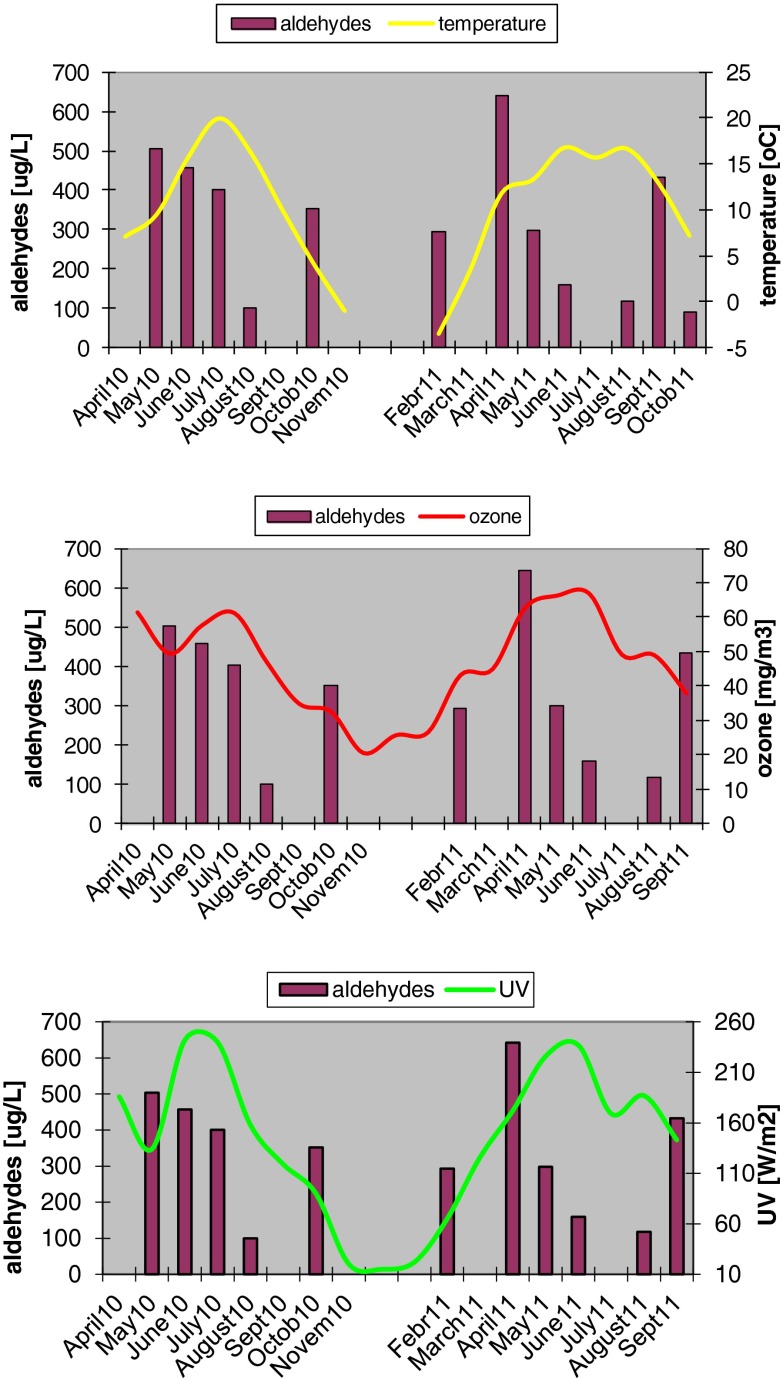
Correlation between atmospheric condition and aldehyde concentration in water of Lake Góreckie

The aldehydes present in the atmosphere can be deposited in the lake water with the precipitations. In order to check the influence of the atmospheric conditions on the content of aldehydes in lake waters, the meteorological data were recorded (Fig. 3), including precipitations, air temperature, ozone concentration and UV radiation intensity. In the period of study, the air temperature varied in a wide range, from −3.5 + 20 °C. The highest mean temperatures were noted in the summer; from June to August, they varied from +16 to +20 °C. Although an increase in some aldehyde concentration has been observed (hexanal, heptanal, nonanal, decanal), no correlation was found however between the air temperatures and total aldehyde concentrations in the lake waters. For instance, the correlation coefficient between the air temperature and aldehyde concentration in Lake Góreckie was about 0 (*r* = 0.04) (Fig. 4).

The UV radiation intensity in Poland is much different in summer and in winter; it is of course the highest in spring and summer. In 2010, there were two characteristic periods of increased UV radiation intensity: in April—to 190 W/m^2^ and in June—to 240 W/m^2^. In 2011, the UV radiation intensity increased in June to 240 W/m^2^, while in July–a period of the greatest precipitations in Poland, so in the most cloudy period—it decreased to 170 W/m^2^, and then in August it increased again to 190 W/m^2^. These distinct changes were not reflected in the level of aldehydes. The highest content of total aldehydes in the near-surface lake water in 2011 was noted in April and not in the period of the greatest UV radiation intensity. The correlation coefficient between the UV radiation intensity and content of total aldehydes in Lake Góreckie was closed to zero.

Ozone—as a strong oxidiser—promotes the formation of carbonyl compounds in the process of ozonation of hydrocarbons commonly present in the atmosphere. The elevated intensity of UV radiation was responsible for increase of ozone content, so these two parameters were strongly correlated and described by *r* = 0.91. As UV radiation intensity is higher in summer, the content of oxygen was also the highest in this season, but no correlation was found between the concentration of ozone in the air and the content of total aldehydes in the near-surface lake water.

**Fig. 5:**
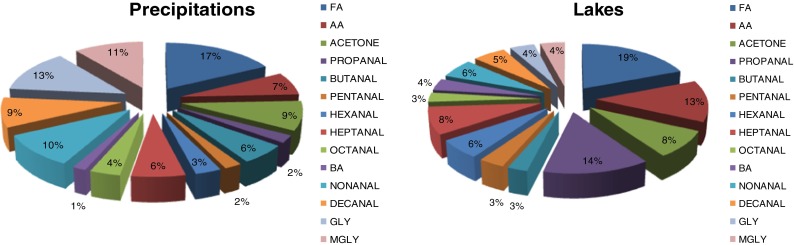
The contributions of monitored aldehydes in the precipitations and lake samples. *FA* formaldehyde, *AA* acetaldehyde, *BA* benzaldehyde, *GLY* glyoxal, *MGLY* methylglyoxal

Water from precipitations is characterised by a high content of aldehydes. The aldehydes present in the air are periodically removed from it by wet deposition and in this way they can get to surface waters. According to our measurements, the concentration of aldehydes in the water from precipitations is higher than that in near-surface lake water. The data supporting this conclusion are presented in Table 5, giving the mean concentrations of aldehydes in the lake water samples and in precipitation water. The latter show much higher mean concentrations of formaldehyde, acetone, nonanal, dekanal, glyoxal and methylglyoxal, commonly occurring in the atmosphere. The contributions of particular aldehydes in the precipitation samples are different from those established in the near-surface lake water (to compare, see Fig. 5. In the water from precipitations, the contributions of formaldehyde, nonanal, dekanal, glyoxal and methylglyoxal are rather high and make over 60 % of all aldehydes. In the surface layers of lake water, the main contribution is brought by aldehydes C1–C3 and acetone, which make over 50 % of all aldehydes.

**Table 5:** Aldehyde concentrations and their frequency of occurrence in precipitation and in lake samples

Compounds	Precipitations (*n* = 30)			Lakes (*n* = 64)		
	Concentration [μg/l]		Frequency of occurrence [%]	Concentration [μg/l]		Frequency of occurrence [%]
	Range	Average		Range	Average	
Formaldehyde	45.2–191	100	100	4.44–121	52.3	100
Acetaldehyde	16.6–177	39.9	100	5.76–112	34.6	100
Acetone	24.8–91.4	50.4	95	3.38–96.2	22.8	100
Propanal	6.69–41.9	12.9	90	6.04–212	37.1	100
Butanal	14.3–83.5	33.3	75	2.00–26.9	7.40	92
Pentanal	5.28–48.9	11.7	75	0.10–40	9.2	75
Hexanal	11.20–84.7	18.2	75	2.34–87.1	16.5	88
Heptanal	15.6–90.2	37.1	60	4.94–113	23.1	55
Octanal	12.2–56.5	21.2	75	2.50–32.9	7.84	30
Benzaldehyde	4.02–41.1	9.11	50	2.18–18.3	9.70	26
Nonanal	16.3–111	56.6	85	1.25–82.9	16.4	81
Decanal	14.1–104	50.6	80	1.93–73.9	15.4	60
Glyoxal	45.5–175	73.6	85	0.10–59	11	85
Methylglyoxal	10.2–90.7	60.8	100	0.11–91	11	82

**Fig. 6:**
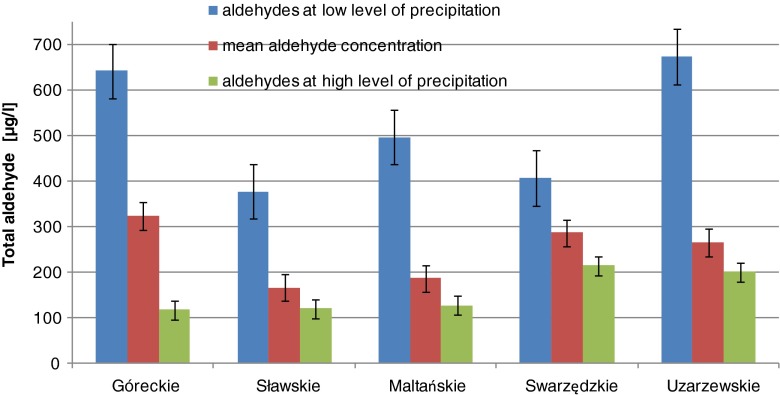
Comparison of total aldehyde concentrations in lake waters monitored during low and high level of precipitations

In the area where all the lakes studied are localised–western Poland—the mean annual precipitations are near 500 mm/m^2^ and their amounts in particular months are much different. The greatest amount of precipitation in Poland is usually noted in July (Fig. 3). In April 2011, the precipitation was very low—of a few millimetres, while in July it was almost 150 mm. In the period of very low precipitations, the highest contents of total aldehydes were determined in all of the water samples; these values were significantly higher than the mean total aldehyde concentration (see the data in Fig. 6). In the periods of intense precipitations, the content of total aldehydes was drastically smaller. The mean concentration of aldehydes in the surface layer of lake water was about 3.5 times higher in the period of low precipitation than that noted in the period of high precipitations; in the water from Lake Góreckie, this difference was even five times. This negative correlation was also reported by Kawamura et al. (2001), who explained this phenomenon by the effect of dilution: at more intense precipitations, the concentration of compounds washed out from the atmosphere is smaller, but at low precipitations, these compounds are more concentrated. We observed that in the first minutes of precipitation, especially after a long dry period, the water from precipitations contain particularly high content of aldehydes, and with increasing time of precipitation, this content decreases, even by a few times. On the basis of the mean total content of aldehydes in rain water which is near 600 μg/l and assuming a mean annual precipitation of 500 mm/m^2^, the amount of aldehydes brought with wet deposition is close to 0.3 g aldehydes per square meter per year. Assuming that the aldehydes brought with rainfall reach lakes only through their surface and stay in the near-surface layer up to a depth of 0.5 m, we can estimate that the content of aldehydes coming from precipitates and present in the surface layer of lakes is 600 μg/l per year, which gives 50 μg/l per month. It makes from 15 to 30 % of the content of aldehydes determined in the surface layer of lakes water; so, theoretically this amount of aldehydes can come from precipitates, while the rest—of over 70 %—seems to originate from the processes taking place in the lakes.

## Conclusions

Two-year examinations conducted in the seasonal cycles in six lakes located in western Poland showed the presence of significant amounts of aldehydes. The contents of total of aldehydes in the surface layer of the lakes varied in a wide range, from 55 to 670 μg/l, and the aldehydes represented include at least the ones from C1 to C10, benzaldehyde, glyoxal and methylglyoxal. Additionally, acetone was commonly detected in the water samples. The concentration of total organic carbon (TOC) also fluctuated significantly, from 3 to 18 mg/l, and the aldehyde contribution to TOC oscillated between 1.32 and 2.88 %. The total content of aldehydes in the surface lake water did not depend on the meteorological parameters such as the air temperature, UV radiation and ozone concentration. In the period of particularly low level of precipitations, the concentration of aldehydes in the surface lake waters was significantly higher than the mean total concentration. On the contrary, in the period of high precipitations, it was lower than the mean total concentration. The percentage contributions of particular aldehydes in the water from precipitations are different from those in the lake water samples. The seasonal appearance of elevated levels of nonanal and decanal in lake water samples was noted. Various concentrations and great dynamics of the composition of aldehydes in the environmental waters are both pointing out to the need for precise monitoring and assessment of the aldehyde effects and the evoked risks on the natural aquatic systems.
